# From Classroom to Cleanroom: Evaluating Industrial Field Visits as a Pedagogical Tool in Parenteral Pharmaceutical Manufacturing and Quality Control Education

**DOI:** 10.3390/pharmacy14020062

**Published:** 2026-04-17

**Authors:** Sandi Ali Adib, Husam M. Younes

**Affiliations:** 1 Tissue Engineering and Nanopharmaceuticals Research Laboratory, Office of Vice President for Research and Graduate Studies, Qatar University, Doha P.O. Box 2713, Qatar; 2 College of Pharmacy, Qatar University, Doha P.O. Box 2713, Qatar; 3 Biomedical Research Center, QU Health, Qatar University, Doha P.O. Box 2713, Qatar; 4 School of Life Sciences, Pharmacy and Chemistry, Kingston University London, Kingston upon Thames, London KT2 7LB, UK

**Keywords:** experiential learning, industrial field trips, pharmaceutical manufacturing, parenteral dosage forms, quality control, pharmacy education, learning outcomes

## Abstract

This study investigates the educational impact of an industrial field visit on the learning experience of second-year pharmacy students at Qatar University. The visit, integrated within the Pharmaceutics II course (PHAR 310), was designed to complement theoretical instruction by providing exposure to real-world pharmaceutical manufacturing and quality control processes, particularly for parenteral dosage forms. A mixed-methods approach was employed using quantitative and qualitative data derived from post-visit questionnaires. Findings indicated that students reported positive perceptions of the experience, with the majority indicating improved understanding of key pharmaceutical manufacturing concepts and strong support for the inclusion of similar activities within the curriculum. Qualitative analysis further suggested that the visit facilitated contextualization of theoretical knowledge, enhanced engagement, and supported early professional awareness. While these findings suggest that structured industrial visits may serve as a valuable complementary educational strategy in pharmacy training, the results should be interpreted with caution due to the small sample size and single-institution design. Further research incorporating larger cohorts, objective learning assessments, and longitudinal evaluation is underway to better establish the educational impact of these interventions.

## 1. Introduction

Preparing pharmacy graduates to competently navigate the scientific, technical, and regulatory dimensions of modern pharmaceutical manufacturing requires educational strategies that integrate foundational scientific instruction with meaningful experiential learning. Traditional didactic approaches and laboratory-based teaching remain indispensable for establishing core knowledge of formulation science, sterile product preparation, and quality assurance principles. Evidence across the health and pharmaceutical sciences consistently demonstrates that experiential and active learning approaches enhance student engagement, knowledge retention, and the development of higher-order cognitive skills by situating theoretical concepts within authentic, practice-oriented contexts [1,2]. Within this framework, structured industrial visit-based learning has emerged as a valuable pedagogical strategy, providing learners with direct exposure to real-world pharmaceutical manufacturing processes and professional practice standards that are not readily reproducible in academic environments. Furthermore, integrating such practice-based experiences into foundational pharmacy courses has been shown to support the application of theoretical knowledge and facilitate early professional identity formation [3,4,5]. Accreditation and professional competencies bodies such as Accreditation Council for Pharmacy Education (ACPE), The Canadian Council for Accreditation of Pharmacy Programs (CCAPP), and National Association of Pharmacy Regulatory Authorities (NAPRA) emphasize the importance of integrating practice-relevant, ability-based education that supports the development of professional competencies, critical thinking, and readiness for contemporary pharmacy roles [6,7,8]. These standards highlight the need for curricula that bridge theory and practice, cultivate problem-solving and decision-making skills, and situate students within realistic professional environments. Industrial field visits directly contribute to these expectations by enabling learners to observe and interpret how theoretical principles, particularly those relating to sterile manufacturing, aseptic operations, Good Manufacturing Practices (GMP), and Quality Control (QC), are operationalized in pharmaceutical production facilities.

Parenteral products are among the most technically demanding dosage forms in pharmaceutical manufacturing. Their formulation, sterilization, aseptic filling, and quality assurance require strict environmental controls, rigorous validation, and highly specialized equipment, making this domain especially challenging for students to comprehend through classroom instruction alone. Many pharmacy students struggle to translate theoretical knowledge and lab-based training of parenteral formulation, pyrogen control, particulate testing, ingredients quantification, sterility assurance, and visual inspection into a coherent understanding of their practical application [9]. Industrial visits, therefore, offer a critical opportunity for pharmacy students to observe these processes directly, contextualize classroom learning, and develop a clearer understanding of how parenteral products are manufactured and tested in compliance with regulatory requirements.

Experiential learning theory offers valuable insight into why industrial visit-based learning produces measurable educational benefits. Kolb’s learning cycle emphasizes four interconnected components: concrete experience, reflective observation, abstract conceptualization, and active experimentation, which collectively promote deeper comprehension and long-term retention. Industrial visits provide the concrete experience required to activate the remaining phases of this cycle, allowing students to reflect on real manufacturing practices, reconceptualize abstract principles discussed in lectures, and subsequently integrate these insights into laboratory work and problem-based learning activities. This experiential grounding has been shown to strengthen confidence, improve professional identity development, and support the acquisition of higher-order competencies [10,11].

Furthermore, contemporary pharmaceutical education increasingly seeks to introduce students to authentic interdisciplinary environments where engineering, regulatory sciences, quality assurance, and industrial pharmaceutics intersect. Industrial field visits expose students to these multidisciplinary workflows, enabling them to appreciate the coordinated roles of production teams, quality assurance units, microbiology laboratories, validation specialists, and regulatory compliance officers. Exposure to this integrated ecosystem helps students internalize GMP culture, professional expectations, and the critical importance of precision, documentation, and accountability throughout the pharmaceutical manufacturing lifecycle.

For institutions dedicated to training future pharmaceutical scientists and industrial pharmacists, such as the College of Pharmacy (COP) at Qatar University (QU), experiential learning opportunities are of considerable educational value. Within this context, the annual one-day industrial visit embedded in the Pharmaceutics II course to Qatar Pharma, the country’s primary and largest parenteral manufacturing facility, provides second-year students with a unique opportunity to gain exposure to specialized pharmaceutical manufacturing processes. These include water-for-injection production, solution compounding, terminal sterilization, aseptic filling, and pharmaceutical quality control practices. Furthermore, this early exposure is complemented by the elective Structured Practical Experiences in Pharmacy (SPEP) program undertaken in the fourth year at the college [12], which aligns with Advanced Pharmacy Practice Experiences (APPE) implemented in other North American-accredited pharmacy programs [13].

More broadly, industrial visits and related experiential learning approaches have been increasingly integrated into pharmacy and pharmaceutical sciences curricula as structured pedagogical strategies to bridge theoretical knowledge with professional practice [9,14,15]. Such experiences allow students to directly engage with pharmaceutical manufacturing environments, quality systems, and regulatory frameworks, thereby enhancing contextual understanding and fostering professional identity development. In parallel, experiential learning models, including site visits, internships, and practice-based placements, are widely regarded as essential educational components, as they provide authentic settings for applying knowledge, developing technical competencies, and integrating cognitive and practical skills. Empirical evaluations, commonly based on student perception surveys, reflective assessments, and competency-based outcomes, consistently report improvements in student engagement, confidence, and perceived relevance of coursework. However, variability persists in implementation and methodological rigor across institutions.

Despite these recognized benefits, the literature remains limited in providing detailed, systematic investigations of industrial visit-based learning, particularly within the specialized context of pharmaceutical sterile manufacturing and parenteral quality control. While active learning strategies such as case-based learning, inquiry-based laboratory modules, and simulation-based instruction have been extensively studied [2,16], there is a clear need for robust empirical research examining the impact of industrial visits on student learning, program-level outcomes, and alignment with accreditation standards in pharmaceutical science education. Collectively, existing evidence supports the value of industrial visits as an important yet underexplored dimension of experiential learning, underscoring the need for more rigorous and longitudinal evaluations.

This study addresses this gap by evaluating students’ perceptions and self-reported learning following participation in a structured industrial visit focused on parenteral pharmaceutical manufacturing and quality control. Particular emphasis is placed on alignment with the standards of ACPE and CCAPP, both adopted by the College of Pharmacy at QU and internationally recognized benchmarks for competency-based education, experiential training, and quality assurance. Although experiential learning is widely recognized as an essential component of pharmacy education, published studies specifically examining the educational impact of industrial visit–based learning remain limited, particularly within the context of sterile manufacturing. By analyzing student feedback and aligning with predefined course learning outcomes, this study provides preliminary insights into the potential role of structured industrial visits in supporting conceptual understanding, contextualizing theoretical knowledge, and fostering early professional awareness. The findings are intended to inform the design and integration of experiential learning activities within pharmaceutics curricula and to contribute to ongoing discussions on aligning educational practices with accreditation expectations, such as those of ACPE and CCAPP. However, given the exploratory nature of the study, the results should be interpreted as indicative rather than conclusive, highlighting the need for further multi-institutional and longitudinal research.

## 2. Materials and Methods

### 2.1. Study Design and Educational Context

This study adopted a cross-sectional, mixed-methods educational research design to evaluate the pedagogical impact of an industrial field visit on pharmacy students’ understanding of pharmaceutical manufacturing and quality control of parenteral dosage forms. The study was conducted as an integral component of the Pharmaceutics II (PHAR310) course at the COP, QU. The industrial visit was intentionally embedded within the course structure to complement didactic lectures and laboratory-based training, thereby reinforcing theoretical concepts through authentic industrial exposure.

### 2.2. Participants and Industrial Visit Structure

The industrial visit was a mandatory educational activity, whereas participation in the post-visit survey was voluntary and anonymous. To ensure optimal learning exposure and logistical efficiency, students were divided into two groups that rotated between pharmaceutical production areas and quality control laboratories. This rotational structure enabled closer interaction with industrial staff. It facilitated close observation and discussion of sterile manufacturing processes, GMP-controlled environments, and quality assurance operations relevant to parenteral dosage forms. For the detailed industrial visit itinerary, please refer to Appendix A.

### 2.3. Survey Instrument and Data Collection

A structured post-visit evaluation survey was administered electronically via the QU online learning management system, Blackboard^®^ Ultra, approximately 2 days after the industrial visit. The timing was selected to allow reflective processing while minimizing recall bias. The survey instrument comprised six closed-ended items, measured on a five-point Likert scale (Strongly Disagree to Strongly Agree), and one open-ended question designed to elicit reflective feedback on perceived benefits and areas for improvement (Appendix B). The Likert-scale items assessed trip organization, adequacy of time allocation, relevance to pharmaceutical dosage forms and parenteral content, overall educational value, and student support for integrating industrial visits into future courses.

The pharmaceutics teaching team developed the survey instrument based on previously published pharmacy education [17] and other relevant disciplines’ [18,19] evaluation tools. Content validity was established through expert review by two faculty members in pharmaceutics and one expert in education, and partially relying on other published studies [20,21].

### 2.4. Quantitative Data Analysis

Quantitative data were analyzed descriptively using percentage distributions for each Likert-scale response category. Given the exploratory and evaluative nature of the study and the small cohort, inferential statistical analyses were not conducted. The descriptive approach allowed the identification of overall trends in students’ perceptions of learning enhancement, curricular relevance, and experiential value.

### 2.5. Qualitative Thematic Analysis of Student Reflections

Qualitative data from the open-ended post-visit survey items were analyzed using thematic analysis, following the six-phase framework described by Braun and Clarke [22,23]. This approach involved systematic familiarization with the dataset, generation of initial codes, organization of codes into candidate themes, iterative review and refinement, and final definition and interpretation of themes. This analytical process enabled the identification of recurring patterns within students’ reflections and provided deeper insight into how the industrial field trip to Qatar Pharma enhanced the integration of theoretical knowledge with real-world pharmaceutical manufacturing practices covered in the Pharmaceutics II course.

To ensure methodological rigor, reflexivity was maintained throughout the analysis. The research team, comprising faculty members in pharmaceutics education, engaged in ongoing critical reflection and discussion to minimize interpretive bias. Analytical decisions were continuously revisited to ensure that theme development remained grounded in participant data. Emergent themes were further interpreted in relation to established educational frameworks, including Kolb’s Experiential Learning Cycle and the Structure of Observed Learning Outcomes (SOLO) taxonomy [24,25,26], allowing for evaluation of learning depth and cognitive integration beyond surface-level perceptions.

During the coding process, initial codes and candidate subthemes were generated inductively from the dataset and subsequently refined through constant comparison and conceptual clustering. In accordance with the principles of reflexive thematic analysis, themes were defined on the basis of conceptual coherence and interpretive relevance rather than frequency alone. This process yielded four overarching themes that provided a comprehensive, analytically robust representation of the data.

### 2.6. Global Benchmarking and Comparative Analysis

A structured, systematically defined benchmarking framework was applied to facilitate a rigorous, transparent, and reproducible comparison with established international pharmacy education models. The framework was guided by predefined analytical criteria, including: (i) the timing and stage of experiential exposure within the curriculum, (ii) the extent and depth of curricular integration, (iii) alignment with internationally recognized accreditation standards (e.g., ACPE and CCAPP), and (iv) evidence of measurable competency-based outcomes. Benchmarking data were derived from recent peer-reviewed literature (2020–2025) in conjunction with authoritative accreditation guidelines, thereby ensuring methodological robustness, validity, and contemporary relevance of the comparative analysis.

## 3. Results

### 3.1. Quantitative Evaluation of Student Perceptions

**Figure 1:**
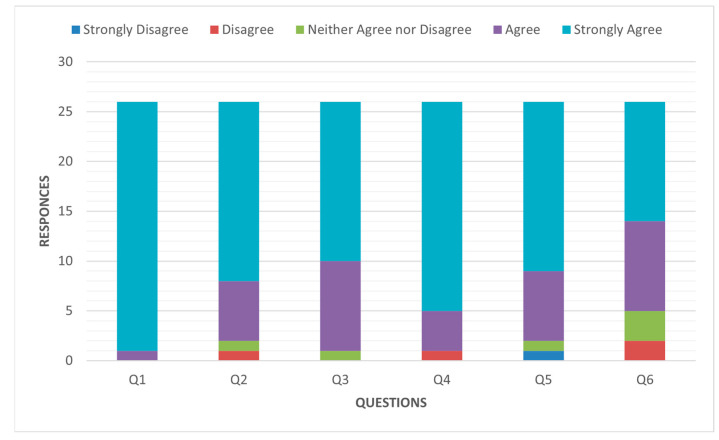
Distribution of Likert-scale responses by question assessing organization, relevance, and educational value.

All second-year pharmacy students enrolled in Pharmaceutics II (n = 26) completed the post-visit survey, yielding a 100% response rate. Overall, students reported highly positive perceptions regarding the organization, relevance, and educational value of the industrial visit to Qatar Pharma. As illustrated in Figure 1, 96.2% of respondents strongly agreed that the visit was well structured in terms of timing, logistics, transportation, and content. In addition, 92.3% of students agreed or strongly agreed that the time allocated to both pharmaceutical production and quality control units was appropriate.

**Figure 2:**
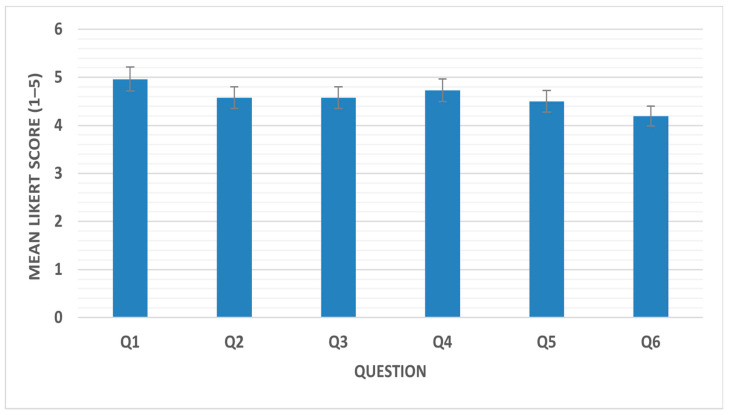
Mean Likert-scale scores across survey items.

Notably, 96.1% of students affirmed that the visit substantially enhanced their understanding of pharmaceutical dosage forms, particularly parenteral preparations. Strong curricular endorsement was evident, with 80.8% of respondents strongly supporting the inclusion of similar industrial visits in future Pharmaceutics III. Furthermore, 46.2% of students strongly recommended integrating factory rotations within the SPEP, with an additional 34.6% agreeing. These findings demonstrate broad student consensus regarding the pedagogical value and curricular relevance of industrial exposure (Figure 2).

**Figure 3:**
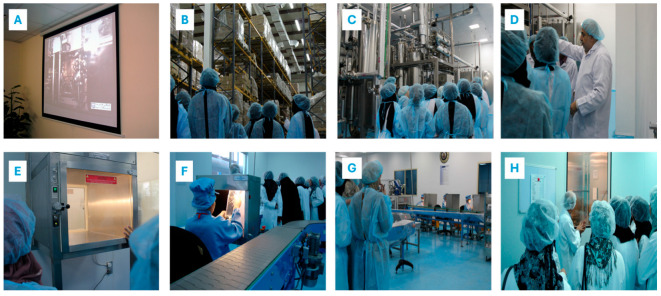
Representative photographic documentation of the structured industrial field visit to a sterile pharmaceutical manufacturing facility, demonstrating progressive student exposure to core unit operations and quality systems in parenteral production. (**A**) Pre-visit orientation session, including a focused lecture and audiovisual reinforcement of sterile manufacturing principles introduced in the theoretical component of the course; (**B**) guided tour of the raw materials and active pharmaceutical ingredients (API) storage and controlled supply area; (**C**) site visit to the Water for Injection (WFI) production and distribution system, with discussion of aseptic utilities and contamination control strategies; (**D**) on-site explanation of Blow–Fill–Seal (BFS) technology, including integrated aseptic forming, filling, and sealing operations and terminal sterilization considerations; (**E**,**F**) supervised exposure to quality control (QC) and microbiological testing laboratories, highlighting environmental monitoring and sterility assurance practices; (**G**) structured interaction with industrial personnel and product inspection teams, emphasizing in-process control and compliance with Good Manufacturing Practice (GMP); (**H**) demonstration of stability chambers and controlled-environment stability testing rooms in accordance with regulatory requirements.

As illustrated in Figure 3, the structured industrial visit provided progressive and focused exposure to sterile manufacturing operations, aseptic utilities, quality control laboratories, and stability testing environments within a regulated GMP framework. The sequential design from pre-visit theoretical reinforcement to on-site engagement with BFS technology, WFI systems, and microbiological testing demonstrates intentional curricular alignment. This experiential immersion facilitated the translation of classroom pharmaceutics concepts into authentic industrial practice and regulatory compliance contexts.

### 3.2. Qualitative Thematic Analysis of Student Reflections

**Figure 4:**
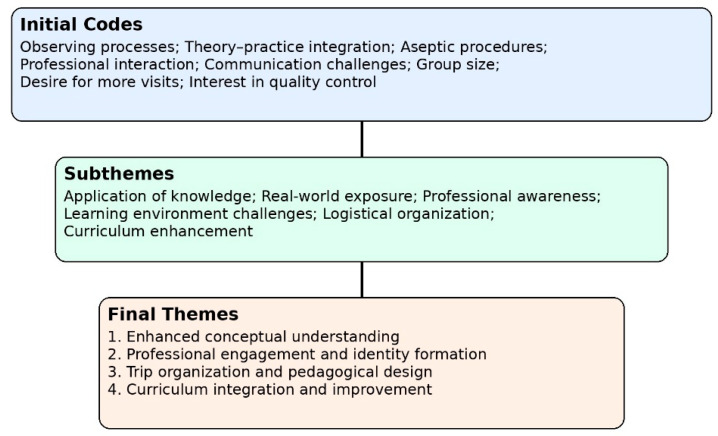
Thematic development process illustrating the progression from initial codes to subthemes and final themes. Codes derived from student reflections were iteratively grouped and conceptually refined into four overarching themes, consistent with reflexive thematic analysis.

Qualitative analysis of student reflections revealed four interrelated themes that complement and contextualize the quantitative findings. The thematic development process, illustrating the progression from initial codes to subthemes and final themes, is presented in Figure 4.

A structured summary of the four main identified themes, representative student quotations, and corresponding quantitative support is provided in Table 1. As reported, the identified themes include: (1) enhanced conceptual understanding, (2) professional engagement and identity formation, (3) trip organization and learning environment, and (4) curriculum integration and improvement.

### 3.3. Global Benchmarking and Comparative Analysis

Comparative benchmarking confirms that experiential education is a core component across global pharmacy curricula; however, its implementation varies substantially in terms of structure, timing, and focus. In North America, experiential learning is primarily delivered through IPPE and APPE frameworks, typically introduced later in the curriculum and predominantly focused on clinical practice [6,8,27]. Similarly, in the United Kingdom, MPharm programs integrate experiential learning aligned with GPhC standards, emphasizing regulatory exposure and reflective learning practices [28]. In Australia and New Zealand, distributed experiential learning models are adopted, often incorporating inquiry-based approaches; however, exposure to pharmaceutical manufacturing remains limited [29]. In contrast, selected Asian models incorporate earlier exposure to pharmaceutical manufacturing and regulatory sciences through structured collaboration with industry and regulatory authorities [30]. In India and comparable pharmacy education systems, industrial exposure is incorporated within undergraduate curricula, often through mandated industrial training and site visits. However, recent evidence indicates that these experiences are frequently observational and lack structured competency-based assessment frameworks, limiting their effectiveness in developing industry-relevant skills [31,32,33]. Collectively, these findings indicate that although experiential learning is globally recognized, early-stage integration of industry-focused exposure, particularly in GMP and pharmaceutical manufacturing, is not systematically implemented. The QU model uniquely integrates early, high-intensity experiential exposure with a strong emphasis on GMP and quality assurance, representing a substantive advancement over existing global approaches.

**Figure 5:**
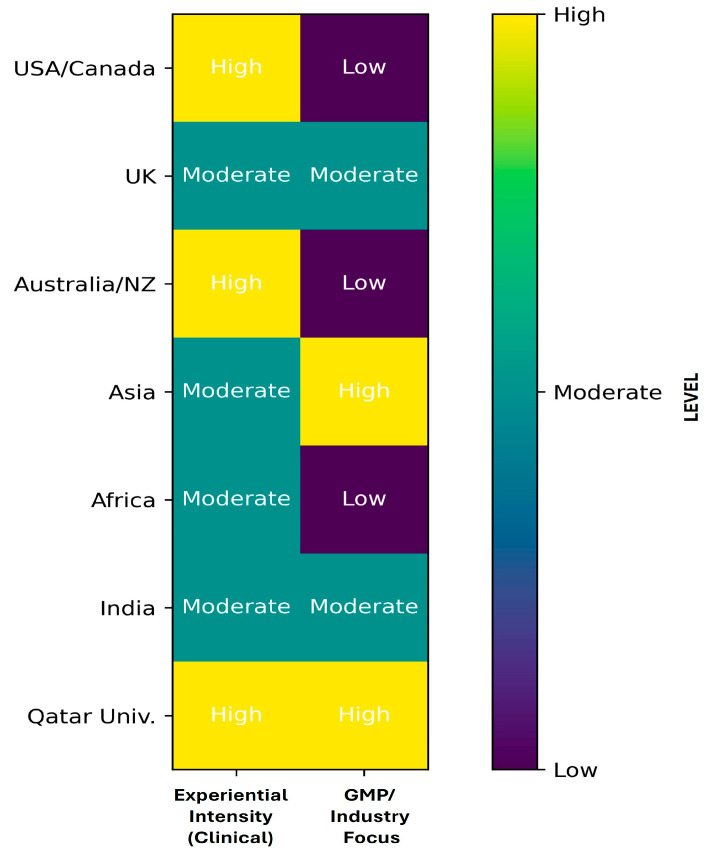
Heatmap comparison of experiential intensity and industry (GMP) focus across global pharmacy education models. A color gradient (Low–High) illustrates the degree of integration.

To enable systematic cross-regional comparison, key benchmarking criteria, including timing of exposure, experiential intensity, and degree of industry (GMP) focus, were evaluated across representative international models (Table 2) and further visualized in a heatmap (Figure 5).

As reported in Table 2, international pharmacy education models generally demonstrate high experiential intensity; however, these experiences are predominantly concentrated in advanced stages of the curriculum and are primarily clinically oriented, with limited emphasis on pharmaceutical manufacturing and GMP-related competencies. In contrast, the QU model uniquely combines high experiential intensity with strong industry and GMP focus at an early curricular stage. This dual advantage enables early contextualization of pharmaceutical manufacturing principles and represents a pedagogical advancement that addresses a recognized gap in global pharmacy education.

The heatmap illustrates the comparative distribution of clinical experiential intensity and industry-focused exposure across global models by using a gradient scale (Low–High). The QU model distinctly demonstrates both high clinical experiential intensity (4th year) and strong industry (GMP) focus at an early curricular stage, differentiating it from other international approaches.

## 4. Discussion

### 4.1. Alignment with Experiential Learning Theory and SOLO Taxonomy

The combined quantitative and qualitative findings align closely with Kolb’s experiential learning cycle. The structured industrial visit served as a concrete learning experience, while post-visit surveys facilitated reflective observation. Students demonstrated abstract conceptualization by synthesizing observed industrial practices with theoretical knowledge, with subsequent application evident in laboratory discussions and assessments.

Analysis of reflective responses further indicated achievement of higher-order cognitive outcomes consistent with the relational and extended abstract levels of the SOLO taxonomy. These findings suggest that industrial visits promote deep, integrative learning beyond surface-level knowledge acquisition.

### 4.2. Qualitative Thematic Analysis of Student Reflections

The findings demonstrate that industrial field visits represent an effective experiential learning approach that enhances conceptual understanding, professional identity formation, and curriculum relevance in pharmacy education. The observed transition from theoretical knowledge to applied understanding aligns with Kolb’s experiential learning cycle, where concrete experience and reflective observation facilitate deeper learning [24,25,26]. Recent studies have similarly demonstrated that experiential and practice-based learning significantly enhances knowledge retention and application compared with traditional didactic approaches [34].

The emergence of professional engagement as a central theme underscores the importance of authentic workplace exposure in supporting early professional identity development. Observing real-world pharmaceutical practice environments enables students to internalize professional roles, responsibilities, and expectations, thereby strengthening the perceived relevance of academic training [35].

Student feedback regarding trip organization highlights the critical role of instructional design in optimizing experiential learning outcomes. Structured facilitation, effective communication, and interactive engagement are essential to ensure that experiential activities move beyond passive observation toward meaningful learning experiences. This aligns with recent pedagogical evidence indicating that experiential learning must be intentionally designed to promote active engagement and critical reflection [36,37]

The strong emphasis on curriculum integration suggests that single exposure, although valuable, may not be sufficient to achieve sustained learning outcomes. Embedding multiple industrial experiences across the curriculum may enhance knowledge retention, support progressive competency development, and strengthen the integration of theory and practice. This perspective is supported by recent advancements in pharmacy education frameworks advocating for longitudinal experiential learning models [8,38].

The consolidation of findings into four overarching themes reflects analytical parsimony and conceptual clarity. Subthemes related to logistical considerations were integrated within broader pedagogical domains to maintain coherence and alignment with theoretical frameworks. This approach enhances interpretability while preserving the depth and richness of qualitative insights.

Collectively, these findings position industrial field visits as a strategically valuable pedagogical approach within pharmacy education. When aligned with accreditation standards such as ACPE and CCAPP, such initiatives can contribute to competency-based education by fostering critical thinking, professional identity development, and practice readiness.

### 4.3. Global Context and Benchmarking

The conducted benchmarking analysis demonstrated that the current model not only aligns with international accreditation standards but also addresses a critical, underrepresented gap in pharmacy education. While global curricula emphasize experiential learning, these experiences are predominantly clinically oriented and introduced at advanced stages, limiting early contextualization of pharmaceutical manufacturing principles. Emerging evidence confirms that early experiential integration enhances knowledge transfer, professional identity formation, and readiness for advanced training [39]. Furthermore, the recent literature highlights a lack of structured industrial experiential models and a persistent gap in preparing pharmacy graduates for industry roles, particularly in GMP, regulatory science, and quality systems [40].

To our knowledge, no prior studies have systematically integrated early-stage, high-intensity, GMP-focused, and structured industrial exposure into foundational pharmaceutics curricula. This positions the present model as a pedagogical innovation with strong potential for scalability and global adoption.

## 5. Conclusions and Future Work

This study supports the educational value of incorporating structured industrial field visits within pharmacy curricula, particularly in pharmaceutical technology and pharmaceutics courses. The findings indicate that students perceived these visits as beneficial in enhancing their understanding of pharmaceutical manufacturing concepts and in contextualizing theoretical knowledge within real-world practice settings. While the results are based on a single cohort, the consistently positive responses suggest that such experiential activities can contribute meaningfully to student learning.

The industrial visit provided students with exposure to key aspects of sterile manufacturing, quality assurance, and regulatory compliance, helping to bridge the gap between classroom instruction and industrial practice. This exposure appeared to support the achievement of course learning outcomes and highlights the potential role of structured site visits as a complementary educational strategy within foundational pharmacy education.

Given the evolving demands of pharmacy practice and industry, integrating experiential learning approaches such as industrial visits may enhance curriculum relevance and student engagement. However, the findings should be interpreted in light of the study’s limitations, including the small sample size, single-institution setting, and primary reliance on descriptive and self-reported data. To build on these preliminary findings, ongoing work is underway to design a more robust research framework that incorporates larger student cohorts, objective learning assessments, and clearly defined competency-based learning outcomes. Such efforts are expected to provide more rigorous evidence on the educational impact and scalability of industrial visit-based learning within pharmacy education.

## Figures and Tables

**Table 1:** Summary of qualitative themes, quotes, and quantitative support.

Theme	Description	Representative Student Reflection *
Enhanced Conceptual Understanding	Students reported improved understanding of pharmaceutical manufacturing concepts through real-world exposure.	I actually observed everything… which enhanced my understanding further.
Professional Engagement and Identity	Exposure to industry professionals strengthened career awareness and professional identity.	Seeing real people working instead of pictures enhanced my perspective.
Trip Organization and Learning Environment	Students evaluated logistics, structure, and communication during the visit.	The trip was very organized and informative.
Curriculum Integration and Improvement	Students suggested incorporating more industrial exposure into the curriculum of other pharmaceutics courses.	Including such tours in other pharmaceutical courses will boost our understanding.

* Refer also to Appendix C for more representative comments.

**Table 2:** Comparative benchmarking of experiential learning models in pharmacy education across global regions, including timing of exposure, experiential intensity, and degree of industry (GMP) focus.

Region	Model	Timing	Experiential Intensity (Clinical)	GMP/ Industry Focus	Strengths	Gap vs. QU
USA/ Canada	IPPE/ APPE	Late	High	Low–Moderate	Strong clinical training	Limited early industry integration
UK	MPharm placements	Mid–Late	Moderate	Moderate	Regulatory integration	Not systematically early
Australia/ NZ	Placements	Distributed	High	Low	Inquiry-based learning	Weak manufacturing emphasis
Asia	Industry-linked	Early–Mid	Moderate	High	Manufacturing exposure	Variable standardization
Africa	Field-based	Mid	Moderate	Low	Applied learning	Limited infrastructure
India	Industrial visits	Early	Low–Moderate	Moderate	Early exposure	Mostly observational
QU	Integrated visit	Early	High	High	GMP + QA integration	Advances the global models

## Data Availability

The original contributions presented in this study are included in the article. Further inquiries can be directed to the corresponding author.

## Abbreviations

The following abbreviations are used in this manuscript:

APC	Australian Pharmacy Council
ACPE	Accreditation Council for Pharmacy Education
API	Active Pharmaceutical Ingredients
APPE	Advanced Pharmacy Practice Experience
BFS	Blow–Fill–Seal
BPharm	Bachelor of Pharmacy
CAPE	Center for the Advancement of Pharmacy Education
CCAPP	Canadian Council for Accreditation of Pharmacy Programs
GMP	Good Manufacturing Practice
COP	College of Pharmacy
GPhC	General Pharmaceutical Council
IPPE	Introductory Pharmacy Practice Experience
MPharm	Master of Pharmacy
NAPRA	National Association of Pharmacy Regulatory Authorities
PBL	Problem-Based Learning
QC	Quality Control
QU	Qatar University
R&D	Research and Development
RTARG	Reflexive Thematic Analysis Reporting Guidelines
SOLO	Structure of Observed Learning Outcomes
SPEP	Structured Practice Experience Program
WFI	Water for Injection

## Appendix A. Field Trip Itinerary

Time Table for the Qatar Pharma Industrial Visit—Pharmaceutics II Course

- 08:00 AM—Departure from Qatar University
- 08:30 AM—Arrival at Qatar Pharma
- 08:45 AM—Welcome briefing by Qatar Pharma staff
- 09:00 AM—Group 1: Production Area Tour/Group 2: Quality Control Lab Tour
- 10:15 AM—Group Swap: Group 2: Production Area Tour/Group 1: Quality Control Lab Tour
- 11:30 AM—Q&A and Debriefing Session
- 12:30 PM—Departure back to campus

## Appendix B. Post-Trip Survey Instrument

The following anonymous questionnaire was administered one week after the industrial field trip to assess student perceptions and learning outcomes.

Please indicate your level of agreement with the following statements:

1. The overall time and transportation for the industrial trip were well structured and planned.
2. The duration of time spent in each segment of the industrial trip was sufficient.
3. The trip complemented my understanding of pharmaceutical dosage forms in general and parenteral dosage forms specifically.
4. Including an industrial trip as part of Pharmaceutics III curriculum (PHAR311) will further strengthen my understanding of pharmaceutics.
5. Overall, I found the industrial trip well-organized and informative.
6. I recommend having a rotation at the Qatar Pharma factory as part of the SPEP rotations.
7. What aspects of the industrial trip did you like, and what aspects would you change? (Short Answers)

## Appendix C. Sample Student Comments

- ‘I liked how I actually observed everything said in the parenteral lecture, which enhanced my understanding further.’
- ‘All parts of the trip were interesting, and they complemented each other. I would not change anything.’
- ‘The visit to the quality control area clarified many concepts discussed in class. I suggest using microphones to hear better.’
- ‘Being exposed to real machinery and staff operations helped me connect theory with practice.’
- ‘I would have liked to hear from my course instructor during the tour to make better links to what we learned.’
